# Crystal structure of putative CbiT from *Methanocaldococcus jannaschii*: an intermediate enzyme activity in cobalamin (vitamin B_12_) biosynthesis

**DOI:** 10.1186/1472-6807-13-10

**Published:** 2013-05-20

**Authors:** Balasundaram Padmanabhan, Shigeyuki Yokoyama, Yoshitaka Bessho

**Affiliations:** 1 Department of Biophysics, National Institute of Mental Health and Neuro Sciences (NIMHANS), Bangalore 560029, India; 2 RIKEN SPring-8 Center, 1-1-1 Kouto, Sayo, Hyogo 679-5148, Japan; 3 Department of Biophysics and Biochemistry, Graduate School of Science, The University of Tokyo, 7-3-1 Hongo, Bunkyo-ku, Tokyo 113-0033, Japan; 4 RIKEN SPring-8 Center, Harima Institute, 1-1-1 Kouto, Sayo, Hyogo 679-5148, Japan

**Keywords:** Cobalamin biosynthesis, Vitamin B_12_, Precorrin 7 C15-methyltransferase, Decarboxylase, CbiT, Crystal structure, AdoHcy cofactor, MJ0391, *Methanocaldococcus jannaschii*

## Abstract

**Background:**

In the anaerobic pathway of cobalamin (vitamin B_12_) synthesis, the CbiT enzyme plays two roles, as a cobalt-precorrin-7 C15-methyltransferase and a C12-decarboxylase, to produce the intermediate, cobalt-precorrin 8.

**Results:**

The primary structure of the hypothetical protein MJ0391, from *Methanocaldococcus jannaschii,* suggested that MJ0391 is a putative CbiT. Here, we report the crystal structure of MJ0391, solved by the MAD procedure and refined to final R-factor and R-free values of 19.8 & 27.3%, respectively, at 2.3 Å resolution. The asymmetric unit contains two NCS molecules, and the intact tetramer generated by crystallographic symmetry may be functionally important. The overall tertiary structure and the tetrameric arrangements are highly homologous to those found in MT0146/CbiT from *Methanobacterium thermoautotrophicum*.

**Conclusions:**

The conservation of functional residues in the binding site for the co-factor, AdoMet, and in the putative precorrin-7 binding pocket suggested that MJ0391 may also possess CbiT activity. The putative function of MJ0391 is discussed, based on structural homology.

## Background

**Figure 1:**
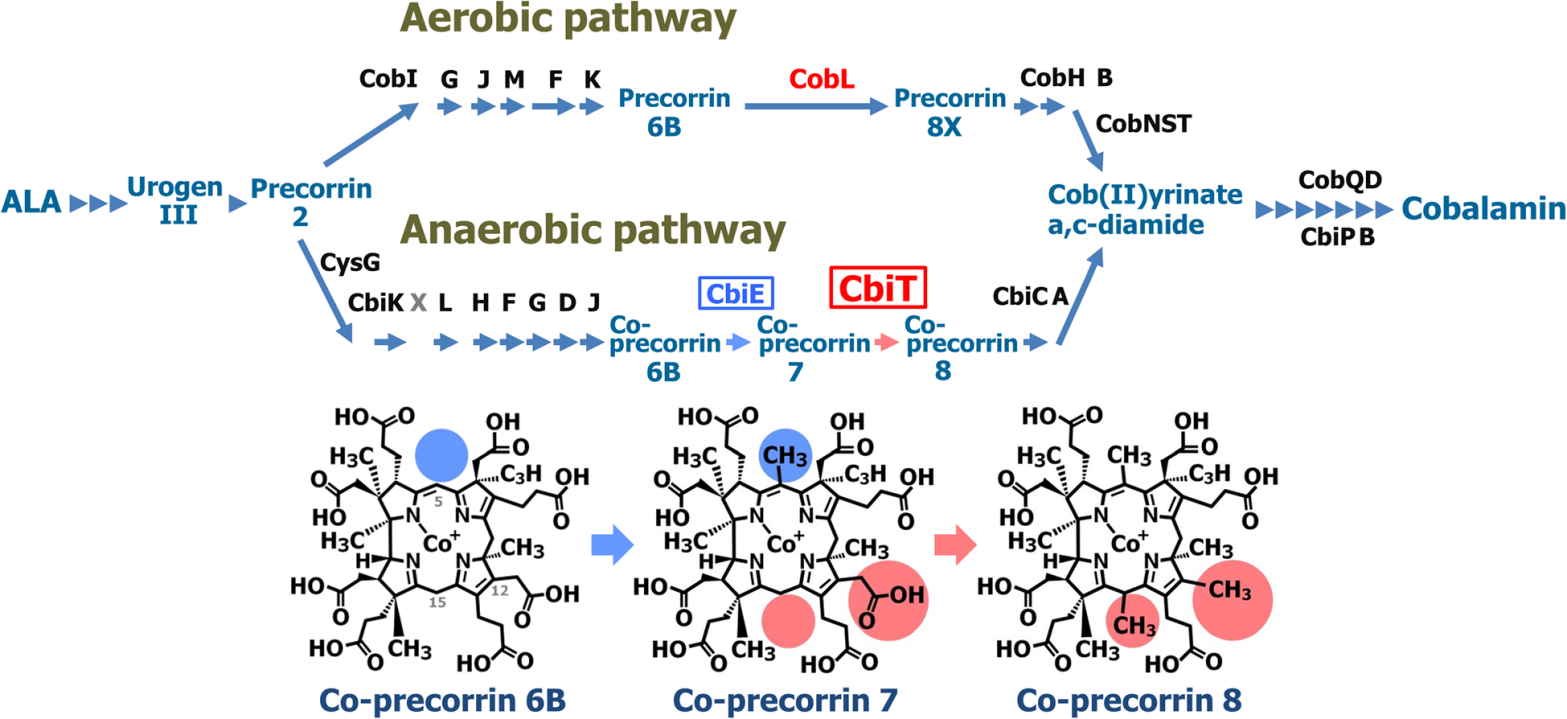
**The aerobic and anaerobic pathways of cobalamin synthesis.** CobL in the aerobic pathway is colored red. CbiE and CbiT, which are equivalent to CobL, are colored blue and red with a boxed label, respectively, in the anaerobic pathway. The chemical structures of cobalt-precorrin-6B, 7 and 8, which are the substrates and products of CbiE and CbiT, are shown at the bottom of the figure.

The *de novo* synthesis of vitamin B_12_ (cobalamin) occurs *via* a complex biochemical pathway involving more than 30 enzyme-mediated steps [1,2]. Therefore, many organisms do not use cobalamin as a coenzyme, and neither synthesize nor require the vitamin in their metabolism. Interestingly, only certain eubacteria and archaea synthesize cobalamin, and the pathway apparently never made the transition to eukaryotes [3]. Although they are genetically distinct, two related pathways are involved in cobalamin biosynthesis in prokaryotes: the aerobic and anaerobic pathways [4]. The aerobic pathway requires molecular oxygen and is characterized by the comparatively late chelation of cobalt into the corrin ring [2,5-8]. In contrast, the anaerobic pathway does not require molecular oxygen, and the cobalt is inserted at an early synthetic stage [4,9,10]. For example, the pathway in *Pseudomonas denitrificans*, an aerobe, incorporates molecular oxygen into the precorrin-3 macrocycle as a prerequisite to ring contraction, and hence is classified as an aerobic pathway. The pathway in *Salmonella typhimurium* synthesizes cobalamin only in the absence of molecular oxygen. This pathway utilizes cobalt ion chelated into precorrin-2 (or Factor II) to set the stage for ring contraction, and hence is classified as anaerobic. The biosynthesis of precorrin-2 is common to both the aerobic and anaerobic pathways. At this stage, the two pathways divide, rejoining only after the synthesis of Cob(II)yrinate *a*,*c* diamide (Figure 1) [1].

The intermediate steps of the aerobic biosynthetic pathway, from uroporphyrinogen III (urogen III) to adenosylcobalamin, have been elucidated by the over-expression of the *cob* genes from *P. denitrificans* (for recent reviews, see [2,11]), and the related assignments of the functions of the encoded enzymes, based on their catalytic activities. The detailed intermediate mechanisms of the anaerobic pathway for cobalamin biosynthesis are progressively emerging, through combinations of molecular genetic, biochemical and three-dimensional structural studies. The *cbi* genes involved in the biosynthesis of cobalamin by the anaerobic pathway have been over-expressed from not only *S. typhimurium* but also *Bacillus megaterium*[12], *Propionibacterium freudenreichi* (*P. shermanii*) [13] and *Methanocaldococcus jannaschii*[14].

The functions of CbiA, -B, -C, -D, -E, -F, -G, -H, -J, -K(X), -L, -P, and -T in the anaerobic pathway were assigned, mainly based on the sequence similarity to their counterparts in the corresponding, well characterized aerobic pathway of *P. denitrificans* (Figure 1). Experimental evidence for the functions of CbiB, -H, -K, and –L, and for those of CbiA, -D, -F, -G, and –T, among the 13 catalytic Cbi proteins, was also recently reported [15-17]. However, only the crystal structures of CbiC [18], CbiF [19], CbiK [20], CbiL [21] and CbiT [22] have been solved.

In the aerobic pathway, the enzyme CobL catalyzes the methylations at C-5 and C-15, and the decarboxylation of the C-12 acetate side chain of precorrin-6B. In contrast, in the anaerobic system, the functions of CobL are shared by two separate enzymes, CbiE and CbiT. Based on the sequence similarity, it has long been assumed that CbiE and CbiT are the methyltransferase and the decarboxylase, respectively: CbiE catalyzes the addition of the two methyl groups to cobalt-precorrin-6B, and CbiT then decarboxylates the C-12 acetate side chain to afford cobalt-precorrin 8. However, recent studies based on structural [22] and biochemical analyses revealed that CbiT possesses both the decarboxylase and methyltransferase activities; i.e., CbiT alone can catalyze both the methylation at C-15 and the decarboxylation of the C-12 acetate side chain, even in the absence of C-1 methylation [16].

The sequence analysis of the hypothetical protein MJ0391, from *M. jannaschii* (hereafter, referred to as MJ0391), suggested that it is closely related to MT0146/CbiT, with 33% sequence identity. Our crystal structure analysis of MJ0391, determined by the multiple anomalous dispersion (MAD) method, suggested that MJ0391 may possess the putative dual functional activities of decarboxylation and methyltransfer in the cobalamin biosynthesis pathway.

## Results and discussion

### Overall structure of MJ0391

**Figure 2:**
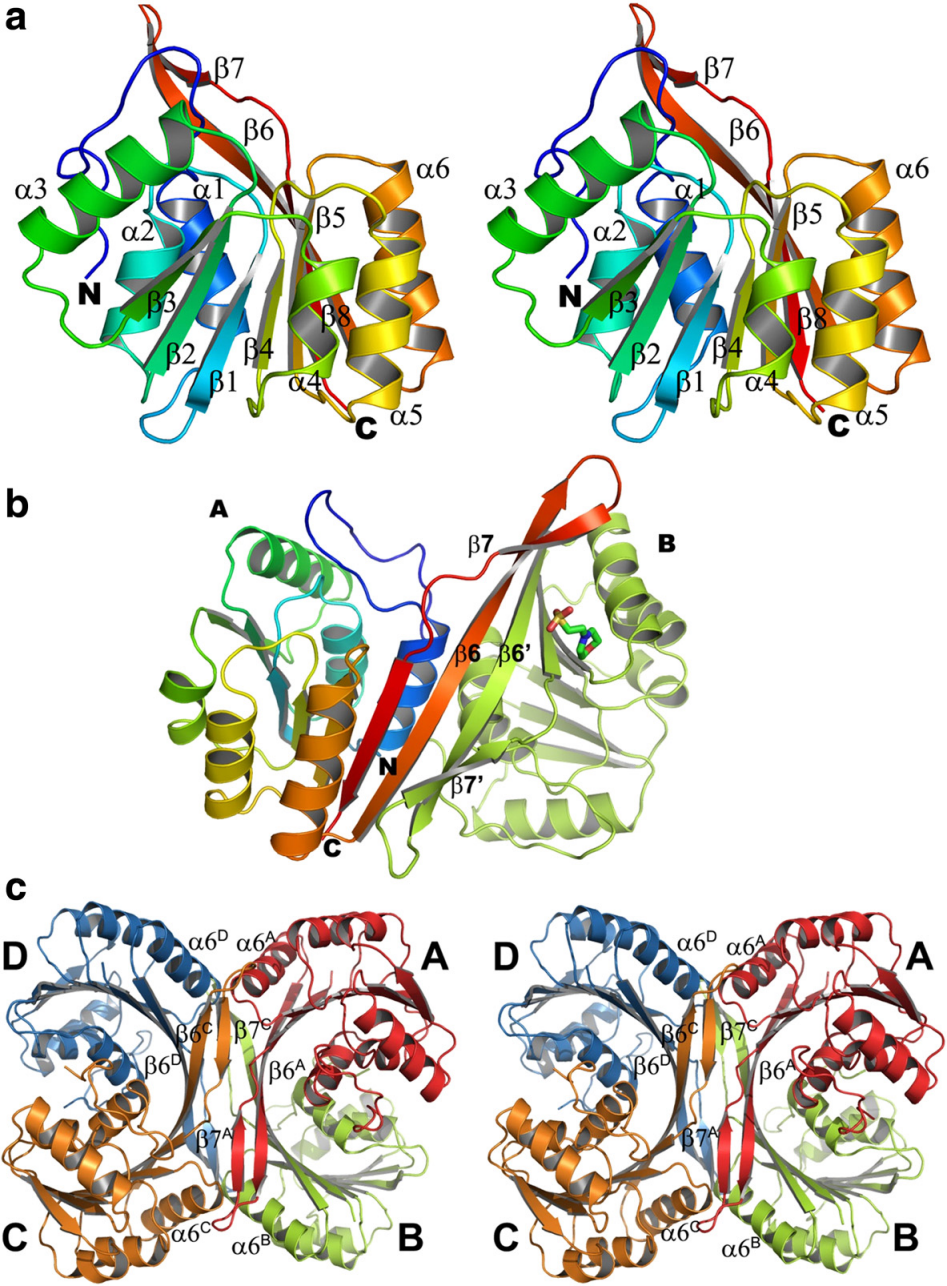
**The structure of MJ0391.** (**a**) Ribbon diagram (stereo view) of the tertiary structure of MJ0391 (colored in a rainbow ramp from blue at the N-terminus to red at the C-terminus). (**b**) The ribbon diagram of the MJ0391 dimer in the asymmetric unit (shown in an arbitrary orientation). Subunit A is colored in a rainbow ramp as in (**a**), and subunit B is colored green. In the dimer, β6 of subunit A and β6’ of subunit B form an intermolecular antiparallel β-sheet. The MES molecule is shown as a stick model. All figures were produced with the *PyMOL* program (Schrödinger, LLC.), unless otherwise mentioned. (**c**) The quaternary structure of MJ0391. Stereo view of the intact tetramer arrangement of MJ0391, produced by the crystallographic 2-fold axis. The tetramer is shown in an arbitrary orientation. Subunits A-D are colored red, green, orange and blue, respectively. A few structural elements at the tetramer interface are labeled, for clarity.

The overall tertiary structure of the MJ0391 protein possesses the Rossmann-like “AdoMet-dependent methyltransferase” fold, comprising seven β-strands (Figure 2a). A long flexible stretch with a non-specific secondary structure conformation is observed at the N-terminal region, Met4 – Thr19. The methyltransferase fold is very similar to a canonical Rossmann fold, but it includes an antiparallel strand (β8) between the parallel strands, β5 and β6, at the C-terminal region. Furthermore, this fold is modified by the extension of the β6 strand, which forms a long irregular β hairpin with the β7 and β8 strands (Figure 2a). The canonical class I Rossmann-like methyltransferase fold consists of a central twisted seven-stranded β-sheet (β3-β2-β1-β4-β5-β8-β6), flanked by two bundles of helices on both sides. All of the strands are parallel except for the β8 strand, which is antiparallel to the other strands. Since the structure of MJ0391 is quite similar to that of MT0146/CbiT [22], the same nomenclature has been used for its description.

### Oligomeric structure

The asymmetric unit contains two MJ0391 molecules (subunits A and B). The two subunits are stabilized by the formation of intermolecular antiparallel β-sheets between β6 and β6’ (Figure 2b). The crystal packing analysis revealed that the MJ0391 protein forms an intact tetramer (dimer of dimers) through the crystallographic 2-fold axis (Figure 2c). Each subunit of the tetramer contributes approximately 27 intermolecular electrostatic interactions to facilitate tetramerization (Table 1). In addition to these direct intermolecular interactions, several indirect hydrogen bond interactions are also formed through buried water molecules at the tetramer interface. The long, irregular β-hairpin, which is formed between the β6 and β7/β8 strands, plays a critical role in the tetramer formation (hereafter, referred to as the tetramerization hairpin (TH) region). The tetramerization occurs due to the contributions of the TH regions of each subunit, A-D. The TH region of subunit A forms intermolecular interactions with subunits B and C (Figure 2c). The β6 strand of subunit A forms an antiparallel sheet with β6’ of subunit B (Figure 2b), while the region connecting the β7 and β8 of subunit A strands forms intermolecular interactions with the corresponding region in subunit C (Figure 2c).

**Table 1:** Potential electrostatic interactions between subunit A and other subunits of MJ0391

**Subunit A**			**Interacting subunits**			**Distance (Å)**
**Chain**	**aa**	**Atoms**	**Chain**	**aa**	**Atoms**	
A	Glu21	OE1	B	Lys30	NZ	3.43
A	Glu22	OE1	B	Lys30	NZ	2.81
A	Lys30	NZ	B	Glu22	OE1	2.78
A	Leu134	N	C	His168	O	2.87
A	Glu135	OE1	C	His168	N	2.85
A	Glu135	OE2	C	His168	ND1	2.64
A	Glu135	OE2	D	Asn142	ND2	3.05
A	Asn142	ND2	D	Glu135	OE2	2.96
A	Glu145	OE1	B	Lys163	NZ	3.46
A	Glu145	OE1	B	His168	NE2	2.9
A	Glu145	OE2	B	Lys163	NZ	3.38
A	Glu145	OE2	B	His168	NE2	2.96
A	Ala153	O	B	Ala161	N	2.86
A	Asn155	N	B	Ser159	O	2.64
A	Asn155	OE2	B	Ser159	N	3.03
A	Asn155	OE2	B	Ser159	O	3.35
A	Phe157	N	B	Phe157	O	2.89
A	Phe157	N	B	Phe157	N	2.84
A	Ser159	N	B	Asn155	O	2.97
A	Ser159	OE2	B	Asn155	N	2.82
A	Ser159	OE2	B	Asn155	O	3.39
A	Ala161	N	B	Ala153	O	3.03
A	His168	N	C	Glu135	OE1	2.85
A	His168	N	C	Glu135	OE2	3.5
A	His168	NE2	B	Glu145	OE1	3.18
A	His168	O	C	Leu134	N	2.87
A	Ala172	O	C	Ala172	O	2.74

An intermolecular twisted β-sheet is formed between β6 and β6’ of subunits A and B, respectively. The residues Ala153, Asn155, Phe157, Ser159, and Ala161 within β6 of subunit A contribute intermolecular main-chain hydrogen bond interactions with the residues Ala161, Ser159, Phe157, Asn155, and Ala153 of subunit B, respectively (Table 1). Moreover, the residues His168 and Ala172 of subunit A, which are located in β7, form main-chain hydrogen bonds with Leu134 (α6) and Ala172 (β7), respectively, of subunit C.

The α1 helix also contacts the corresponding helix of its dimer counterpart. The subunit A residues Glu21 and Glu22 form potential salt links with Lys30 of subunit B. An additional salt link is also observed between Lys30 of subunit A and Glu22 of subunit B (Table 1). The α6 helix of subunit A potentially interacts with subunits B, C and D (Figure 2c). In subunit A, Glu135 electrostatically interacts with His168 and Asn142 of subunits C and D, respectively, while Asn142 of subunit A forms a salt-link with Glu135 of subunit D. Moreover, the Glu145 residue of subunit A also electrostatically interacts with Lys163 and His168 of subunit B. Besides these electrostatic interactions, about 17 residues of subunit A contribute substantial hydrophobic interactions toward tetramer formation (Table 2). The homologous protein MT0146/CbiT forms a tetramer in the crystal structure and in solution [22]. The buried surface area per monomer within the intact tetramer is about 1,907 Å^2^ (~21% buried surface area), as calculated by the program *SURFACE* in the *CCP*4 suite. Since the overall tetramer arrangement in the MJ0391 structure is very similar to that found in MT0146/CbiT, we speculate that in solution, MJ0391 may also form a tetramer that is functionally important for its biological activity.

**Table 2:** Hydrophobic interactions between subunit A and other subunits of MJ0391

**Subunit A**		**Interacting subunits**	
**chain**	**aa**	**chain**	**aa**
A	Ala25	B	Ala25
A	Ile132	C	Ile164
A	Ile132	C	Met169 (Mse169)
A	Val133	C	His168
A	Val133	C	Ser166
A	Val133	C	Gly167
A	Leu134	D	Ile141
A	Leu134	D	Leu134
A	Leu134	D	Ala138
A	Ala138	D	Leu134
A	Ile141	B	His168
A	Val154	B	Ser159
A	Val154	B	Tyr160
A	Val154	B	Glu22
A	Val156	B	Phe157
A	Phe157	C	Phe157
A	Ile158	B	Asn155
A	Tyr160	B	Val154
A	Ser166	C	Val133
A	Gly167	C	Val133
A	Met169 (Mse169)	C	Ile132
A	Met169 (Mse169)	C	Asn174
A	Phe170	B	Asn155
A	Phe170	C	Asn174
A	Ala172	C	Phe157
A	Asn174	C	Met169 (Mse169)

### Comparison with homologous structures

**Figure 3:**
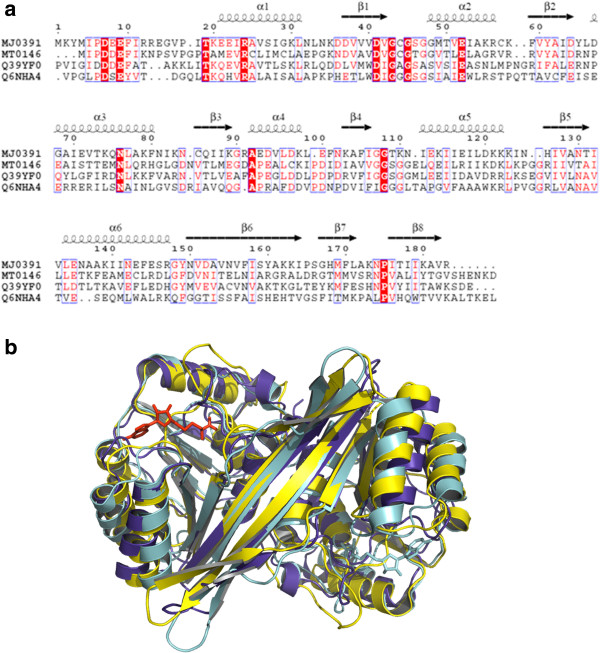
**Comparison with CbiT homologous proteins.** (**a**) Sequence alignment of MJ0391 from *Methanocaldococcus jannaschii*, MT0146/CbiT from *Methanobacterium thermoautotrophicum*, precorrin-6Y C5, C15 – methyltransferase from *Geobacter metallireducens* GS-15 (Q39YF0), and the putative precorrin-6Y C5, C15 – methyltransferase from *Corynebacterium diphtheriae* (Q6NHA4). The secondary structural features from MJ0391 are indicated above the alignments. The colors reflect the similarity (red boxes and white characters for conserved residues; red characters for similarity in a group; blue frames for similarity across groups). The sequence was aligned and rendered by Clustal W [25] and ESPript [26], respectively. (**b**) Superposition of MJ0391 (yellow) on the precorrin-6Y C5, C15 – methyltransferase structures from *G. metallireducens* (PDB ID: 3E05, blue) and CobL^c^ from *R. capsulatus* (PDB ID: 3NJR, cyan).

A DALI [23] search revealed that although MJ0391 shares less sequence similarity with MT0146/CbiT from *Methanobacterium thermoautotrophicum* (33% identity; Z-score: 27.3), the precorrin-6Y C5, C15 – methyltransferase from *Geobacter metallireducens* GS-15 (Q39YF0; 32% identity; Z-score: 26.0), and the putative precorrin-6Y C5, C15 – methyltransferase from *Corynebacterium diphtheriae* (Q6NHA4; 17% identity; Z-score: 17.9) (Figure 3a), the overall tertiary structures and the quaternary structure arrangements are essentially conserved between them. The superimposition of the MJ0391 structure onto the structures of MT0146/CbiT (PDB ID: 1L3I), Q39YF0 (PDB ID: 3E05; 211-405aa), and Q6NHA4 (PDB ID: 3HM2; 236-410aa) yielded r.m.s.d. values of 1.6, 1.7, and 1.9 Å, respectively, for all C^α^ atoms (Figure 3b). Recently, the crystal structure of the C-terminal region of CobL (CobL^c^) from *Rhodobacter capsulatus* was reported [24]. The enzyme CobL, which uses precorrin-6B as a substrate, promotes the decarboxylation of the acetic acid side chain at C12, and the methylation at the C5 & C15 *meso* positions, to produce precorrin-8X. The superimposition of MJ0391 onto the CobL^c^ structure (PDB ID: 3NJR) yielded Z-score and r.m.s.d. values of 25.6 and 1.8 Å, respectively, for 173 C^α^ atoms (Figure 3b).

As expected, the DALI search with MJ0391 also identified proteins belonging to the methyltransferase family. For example, MJ0391 is structurally homologous to m^1^A58 tRNA methyltransferase, TrmI (PDB ID: 1O54, Z-score: 20.6, 2.9 Å r.m.s.d. for 174 C^α^ atoms, 12% identity), m^5^U1939 23S rRNA methyltransferase, RlmD (PDB ID: 2BH2, Z-score: 20.0, 2.6 Å r.m.s.d. for 169 C^α^ atoms, 16% identity), catechol O-methyltransferase, COMT (PDB ID: 1JR4, Z-score: 17.8, 2.3 Å r.m.s.d. for 161 C^α^ atoms, 17% identity), and rRNA dimethyladenosine transferase, Dim1 (PDB ID: 3FYC, Z-score: 18.7, 2.4 Å r.m.s.d. for 153 C^α^ atoms, 30% identity).

### Comparison with MT0146/CbiT

**Figure 4:**
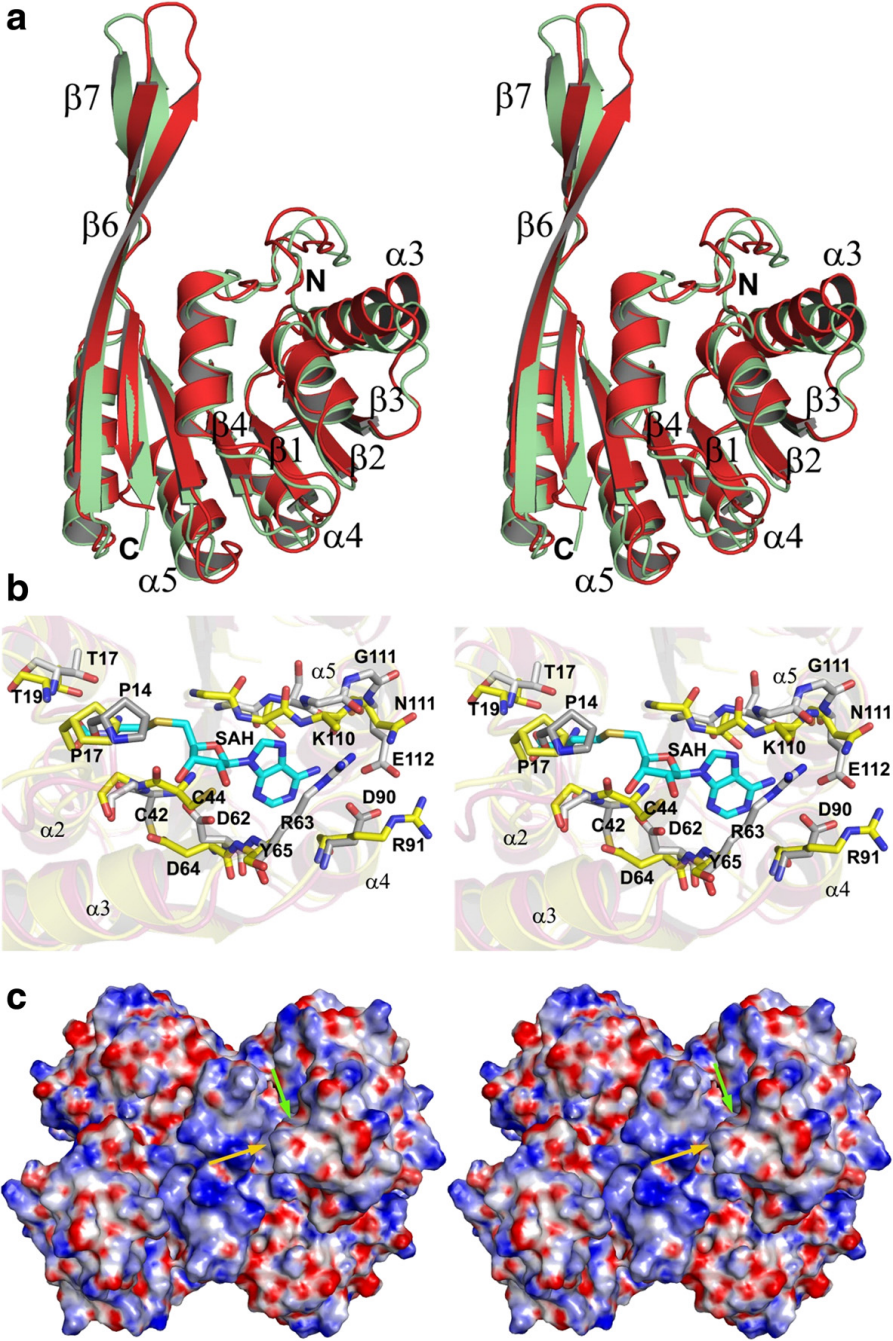
**Comparison of MJ0391 with MT0146/CbiT.** (**a**) Stereo view of the superposition of MJ0391 (light green) on MT0146/CbiT (red), for all C^α^-atoms corresponding to one subunit of the MT0146/CbiT tetramer (PDB ID: 1L3I). (**b**) The AdoHcy binding site region (stereo view). The interacting residues and the AdoHcy molecule (shown as ‘SAH’ in the figure) of the MT0146/CbiT complex are colored grey & cyan, respectively. The equivalent predicted interacting residues in MJ0391 are yellow. The Tyr65 residue is modeled as Ala, since the electron density corresponding to its side chain was not visible. (**c**) Electrostatic surface potential of the MJ0391 tetramer. The surfaces are colored blue and red for positive and negative electrostatic surface potentials, denoting less than -15 and greater than +15 *K*_b_T, respectively, where *K*_b_ is the Boltzmann constant and T is the temperature. The yellow and green arrows indicate the putative precorrin and AdoMet binding pockets, respectively. The surface potential was calculated with the software *APBS*[27] incorporated in the *PyMOL* program (Schrödinger, LLC.).

Although the overall tertiary structures of the MJ0391 and MT0146/CbiT proteins are essentially similar, striking differences exist in the TH region, an important element for forming the tetrameric arrangements (Figure 4a). The dihedral angles at the equivalent positions of Lys163 (φ, ψ: -68, 140°) and Ala166 (φ, ψ: -69, 81°) in MJ0391 and MT0146/CbiT, respectively, trigger large deviations in their respective TH elements. Moreover, the β6 & β7 strands in MJ0391 are connected by a type II β-turn. The type II conformation is produced by incorporating Pro165 at the i+1 position. The β-turn is stabilized by an intra-hydrogen bond between the carbonyl-group of Ile164 (i position) and the amino-group of Gly167 (i+3 position). In contrast, in MT0146/CbiT, β6 and β7 are connected by a relatively long loop, comprising six amino acids. The abovementioned structural deviations cause the maximum r.m.s.d value of 6.2 Å between the C^α^ position of Pro165 in MJ0391 and that of Asp168 in MT0146. The α6 helix also participates in tetrameric interactions (Figure 2c); however, less structural deviation is observed between the corresponding regions of MJ0391 and MT0146/CbiT. In addition, deviations were observed in the non-specific secondary structure regions, such as the N-terminal region and the loops connecting the secondary structural elements (Figure 4a).

A comparison of the apo-form MJ0391 to the MT0146/CbiT complexed with AdoHcy (SAH molecule, S-Adenosyl-L-Homocysteine; PDB ID: 1L3I) [22] revealed that the co-factor binding site regions are similar in both structures (Figure 4b). Hence, we predict that the MJ0391 protein may also recognize the cofactor AdoMet in a similar manner as in MT0146/CbiT. The shallow binding pocket is produced by the head regions of β1, β2 and β4, which line the middle of the pocket. The pocket is surrounded by the α2 and α3 helices on one side, and the α4 and α5 helices on the other side (Figure 4b). The residues within the loops connecting β4 and α5, β2 and α3, β3 and α4, β1 and α2 and in the N-terminal flexible region may potentially form interactions with the AdoHcy molecule. The AdoHcy interacting residues are nearly conserved between these proteins, except for the residues Arg91, Tyr65, Lys110 and Asn111, which are replaced by Asp90, Arg63, Gly111 and Glu112, respectively, in MT0146/CbiT. These loops are quite flexible, as observed in Figure 4a, and thus we speculate that they may undergo conformational changes upon binding to the AdoHcy/AdoMet molecule.

Near the AdoMet binding site, a deep groove is formed by segments from the Rossmann-like methyltransferase core and the TH region (of the same subunit and of the adjacent subunit) (Figure 2c). Keller *et al*. [22] suggested that this deep groove is a putative binding pocket for the precorrin molecule. Surface potential analyses revealed that the MJ0391 protein is highly basic (theoretical pI: 9.01) (Figure 4c), whereas its homologous protein, MT0146/CbiT, is highly acidic (theoretical pI: 4.88). Although the electrostatic charge distributions are quite different between these two proteins, the charge distributions within the putative precorrin binding pocket are highly conserved between them (Figure 4c). The residues Pro17, Arg24, Lys162, Lys173 and Asn174 are located near the binding pocket in MJ0391. The equivalent corresponding residues in MT0146/CbiT are Pro14, Arg22, Arg165, Arg176 and Asn177, respectively (Figure 3). Recent studies of *S. typhimurium* CbiT revealed that CbiT alone can catalyze both the methylation at C15 and decarboxylation of the C12 acetate side chain, even in the absence of C1 methylation [16]. Since MJ0391 is structurally highly homologous to the known CbiT proteins, and the functional residues are conserved between them, we speculate that MJ0391 may also possess the dual roles of methylation and decarboxylation to form the intermediate, cobalt-precorrin 8, in the vitamin B_12_ biosynthesis pathway. However, further biochemical and crystallographic studies are required to understand the enzymatic mechanism of MJ0391 in cobalamin synthesis.

## Conclusions

The structure of the hypothetical protein MJ0391, from *M. jannaschii*, was determined by the MAD procedure, which revealed the presence of a Rossmann-like “AdoMet-dependent methyltransferase” fold. The tertiary and quaternary structures of MJ0391 are essentially similar to those of the CbiT or CobL^c^ proteins *from M. thermoautotrophicum, G. metallireducens,* and *R. capsulatus*. Moreover, the functional residues in the putative precorrin binding pocket are highly conserved. Hence, based on structural comparison studies, we speculate that the MJ0391 protein may possess the putative dual C15-methyltransferase and C12-decarboxylase activities in the cobalamin biosynthesis pathway.

## Methods

### Protein expression and purification

The gene encoding the MJ0391 protein (gi:15668567) was amplified *via* PCR using *Methanocaldococcus jannaschii* DSM 2661 genomic DNA, and was cloned into the pET-21a expression vector (Merck Novagen, Darmstadt, Germany). The expression vector was introduced into the *E. coli* BL21-CodonPlus (DE3)-RIL-X strain (Stratagene, La Jolla, California, USA). The recombinant strain was cultured in 3 L minimal medium, containing 25 μg/mL selenomethionine, 30 μg/mL chloramphenicol and 50 μg/mL ampicillin. The harvested cells (10.7 g) were lysed by sonication on ice, in 20 mL of 20 mM Tris–HCl buffer, pH 8.0, containing 500 mM NaCl, 5 mM β-mercaptoethanol and 1 mM phenylmethylsulfonyl fluoride. The cell lysate was heat-treated at 363 K for 11.5 min and centrifuged at 14,000 g for 30 min at 277 K. The supernatant was desalted by fractionation on a HiPrep 26/10 column (GE Healthcare Biosciences) with 20 mM Tris–HCl buffer, pH 8.0. The fractions of interest were then applied to a Super Q Toyopearl 650M column (Tosoh, Tokyo) equilibrated with 20 mM Tris–HCl buffer, pH 8.0, and eluted with a linear gradient of NaCl. The target fractions, which eluted in 0.1 M NaCl, were applied to a Resource Q column (GE Healthcare Biosciences) equilibrated with 20 mM Tris–HCl (pH 8.0). The flow-through fraction was collected and applied to a Hydroxyapatite CHT-I column (Bio-Rad Laboratories), equilibrated with 10 mM potassium phosphate buffer (pH 7.0), and eluted with a linear gradient of potassium phosphate. The target sample, which eluted in 207.2 mM potassium phosphate, was collected and applied to a HiLoad 16/60 Superdex 200pg column (GE Healthcare Biosciences), equilibrated with 20 mM Tris–HCl buffer, pH 8.0, containing 200 mM NaCl. The protein sample was analyzed by SDS-PAGE and was confirmed by N-terminal amino-acid sequencing. After concentration to 27.9 mg/mL by ultrafiltration, the protein yield was 11.7 mg from 10.7 g of cells.

### Crystallization and data collection

Crystallization screening was performed by the sitting-drop vapor-diffusion method at 293 K. Each drop consisted of 1.0 μL of the Se-Met protein (28 mg/mL) solution and 1.0 μL of the reservoir solution, and was equilibrated against 100 μL of reservoir solution. In the preliminary screening, small crystals were observed in the crystallization conditions of 40%(*v*/*v*) MPD, 0.1 M Na HEPES, pH 7.0. After optimization, crystals suitable for X-ray data collection appeared in 50%(*v*/*v*) PEG-400, 0.1 M MES buffer, pH 6.0, within 2 months. The crystals were flash-cooled in a nitrogen-gas stream at 100 K, using 25%(*v*/*v*) glycerol as a cryoprotectant.

Anomalous diffraction data were collected at the SPring-8 (Harima, Japan) BL26B1 beamline, at three wavelengths, 0.97891 (peak), 0.97928 (edge) and 0.90000 Å (remote), for a Se-MAD experiment. The data sets were collected at 100 K, using a Rigaku Jupiter 210 CCD detector. The crystal-to-detector distance was set to 200 mm. The diffraction data were integrated, reduced, and scaled using the *HKL2000* software suite [28]. The crystallographic data and refinement statistics are shown in Table 3. The asymmetric unit consists of two NCS molecules, with a molecular mass of 21 kDa and 183 amino acid residues per monomer, and with a solvent content of about 42%.

**Table 3:** Summary of data collection, MAD structure solution, and refinement statistics

	**Se-Met peak**	**Se-Met inflection**	**Se-Met remote**
***Data collection***			
Wavelength (Å**)**	0.97891	0.97928	0.90000
Space group	*P*4_1_2_1_2		
Unit cell (Å)	a = b = 93.44, c = 81.04		
Resolution (Å) ^1^	50.00–2.2 (2.28–2.20)		
Completeness (%)	96.6 (78.7)	98.6 (89.6)	99.3 (93.4)
Redundancy	12.2 (8.3)	12.8 (9.4)	13.0 (9.2)
Average I / σ(I)	27.4 (1.9)	30.3 (2.2)	33.7 (2.3)
R_merge_ (%)^2^	9.1 (42.0)	7.9 (43.3)	7.6 (46.9)
***MAD solution***			
Resolution cutoff (Å**)**	2.5		
No. Se-sites found	6 (of 8)		
Mean FOM	0.62		
Overall Z-score	24.3		
***Refinement statistics***			
No. molecules in a.u.	2 molecules		
Resolution limit (Å)	20–2.3		
R-factor/R_free_ (%)^3,4^	19.8 / 27.3		
Mean B factor (Å^2^)	49.0		
No. protein residues	360		
No. MES molecules	1		
No. SO_4_ molecules	2		
No. water molecules	108		
*r.m.s. deviations*			
Bond lengths (Å)	0.013		
Bond angles (^o^)	1.54		

^1^Numbers in parentheses are values in the highest resolution shell.

^2^R_merge_ = Σ |Iobs - <I>| / Σ <I> summed over all observations and reflections.

^3^R_cryst_ = Σ ||F_obs_| – |F_calc_|| / Σ |F_obs_|.

^4^R_free_ was calculated with 5% of data omitted from refinement.

### Structure determination and refinement

The structure determination was performed by the multiple anomalous dispersion (MAD) method, using *SOLVE/RESOLVE*[29]. The MAD procedure yielded six distinct peaks, corresponding to six of the eight selenium atoms in the asymmetric unit, with an overall experimental mean figure of merit of 0.62 for all data up to 2.5 Å resolution. The initial experimental phases were further improved by solvent flattening with 2-fold non-crystallographic symmetry (NCS) averaging, using the program *RESOLVE*[30], which increased the figure of merit to 0.71. The modified experimental map obtained by *RESOLVE* was readily interpretable, and 42% of the protein chains were traced automatically. Moreover, several rounds of manual fitting and re-fitting were performed using the program *COOT*[31]. Initially, the model was refined with *CNS*[32], using a maximum likelihood target that included amplitude and phase probability distributions with NCS restraints. In the later stage of the refinement cycles, the refinement was performed using *REFMAC5*[33], incorporated in the *CCP*4 package [34]. The final refined model of the dimer in the asymmetric unit contains 360 residues, one MES molecule, two SO_4_^2-^ molecules, and 108 water molecules, with a final *R*_work_ of 19.8% and an *R*_free_ of 27.3% at 2.3 Å resolution. The first three N-terminal residues of the two chains are missing in the final structure. The side chain atoms of Mse4, Arg12, Arg13, Tyr65, Leu66 and Lys110 in chain A, and Mse4, Arg12, Arg13, Tyr65 and Lys110 in chain B are also missing in the final structure. The stereochemistry of the complex structure is good, as assessed with *MOLPROBITY*[35,36]. The Ramachandran plot analysis of this structure revealed that 99.4% of all residues were in the allowed region. The outlier residues are Thr109 and Asn174 in chain A. The electron densities were clear within these disallowed regions. The refinement statistics are summarized in Table 3.

### PDB Reference

The structural coordinates of MJ0391 have been deposited in the RCSB (PDB code: 2YXD).

## Abbreviations

NCS: Non-crystallography symmetry; MAD: Multiple anomalous dispersion; r.m.s.d.: Root mean square deviation; CobLc: C-terminal region of CobL.

## Competing interests

The authors declare that they have no competing interests.

## Authors’ contributions

BP, SY and YB coordinated and designed the research work. BP performed the data processing and crystallography studies, and prepared the manuscript. YB performed the protein production and crystallization, and participated in drafting the manuscript. All authors read and approved the final manuscript.
